# Effect of Photoreduction of Semiconducting Iron Mineral—Goethite on Microbial Community in the Marine Euphotic Zone

**DOI:** 10.3389/fmicb.2022.846441

**Published:** 2022-04-11

**Authors:** Jia Liu, Xiao Ge, Hongrui Ding, Shanshan Yang, Yuan Sun, Yanzhang Li, Xiang Ji, Yan Li, Anhuai Lu

**Affiliations:** Beijing Key Laboratory of Mineral Environmental Function, The Key Laboratory of Orogenic Belts and Crustal Evolution, School of Earth and Space Sciences, Peking University, Beijing, China

**Keywords:** goethite, ferrous ions, photoreduction, microbial community, marine euphotic zone

## Abstract

Marine euphotic zone is the pivotal region for interplay of light-mineral–microorganism and elements cycle, in which semiconducting minerals exist widely and iron-bearing goethite is a typical and widespread one. In this work, we have conducted in-depth researches on the effect of ferrous [Fe(II)] ions dissolved by photoreduction of goethite on microbial community structure and diversity. The mineral phase, structure and morphology of synthesized goethite were characterized by Raman, X-ray diffraction (XRD), energy disperse spectroscopy (EDS), environmental scanning electron microscope (ESEM), and atomic force microscope (AFM). Photoelectrochemical measurements tested photoelectric response and redox activity of goethite, having proved its significant property of photoelectric response with 44.11% increment of the average photocurrent density relative to the dark current density. The photoreduction experiments of goethite were conducted under light condition in simulated seawater. It has suggested the photoreduction of goethite could occur and Fe(III) was reduced to Fe(II). The dissolved Fe(II) from the photoreduction of goethite under light condition was nearly 11 times than that group without light after a 10-day reaction. Furthermore, results of microbial community sequencing analysis indicated that dissolved Fe(II) could affect the structure and regulate the decrease of microbial community diversity. The emergence of dominant bacteria associated with iron oxidation and transport protein has suggested their obvious selectivity and adaptability in the environment with adding dissolved Fe(II). This work revealed the photoreduction process of semiconducting goethite was remarkable, giving rise to a non-negligible dissolved Fe(II) and its selective effect on the structure, diversity, as well as the function of microbial community. This light-induced interaction between minerals and microorganisms may also further regulate correlative metabolic pathways of carbon cycle in the marine euphotic zone.

## Introduction

In nature, semiconducting minerals can absorb the energy of solar photon, so that electrons in molecular orbitals leave the valence band and excite to the conduction band, where photo-holes and photoelectrons form, respectively (Lu et al., 2004; Li et al., 2006). There was evidence in favor of a novel pathway, in which solar energy is converted to chemical energy by photocatalysis of semiconducting minerals to support and stimulate the growth of non-phototrophic microorganisms, and help shaping microbial communities (Lu et al., 2012a; Ding et al., 2014; Ren et al., 2018b,^2019^). In the process of microbial growth and metabolism with photoelectrons of semiconducting minerals, the generated photoelectron directly or indirectly participates in the microbial electron transfer (Lu et al., 2012b; Ren et al., 2018a). Under light irradiation, semiconducting minerals, like goethite, rutile, sphalerite produced photoelectrons have been reported to successfully support the growth of chemoautotrophic *Acidithiobacillus ferrooxidans*, heterotrophic *Alcaligenes faecalis*, and the change of natural soil microbial community (Lu et al., 2012b; Wang et al., 2015).

The marine euphotic zone, as one of the most vital parts of the hydrosphere environment on earth, is a critical zone for the intersection of land and ocean. It also involves the interaction between human activities and natural processes, bringing about widespread and abundant microbial communities with active metabolic processes (Zhuang et al., 2019; Liu Y. et al., 2020; Chen et al., 2021). In typical hydrosphere habitats, microorganisms mediate and drive the cycle of important geochemical elements, such as carbon, nitrogen, and sulfur (Lai et al., 2020; Xu et al., 2020; Zheng et al., 2020; Xie et al., 2021). In turn, microbial metabolism and metabolic pathways are impacted by biogeochemical elements cycle and availability, such as iron element.

Iron, as one of the most abundant elements in the earth’s crust, presents both ferrous [Fe(II)] and ferric [Fe(III)] states as ionic species in the marine. It is an essential transition metal for growth and survival of most living organisms, especially for microorganisms. This transition metal is interconverted between its Fe(II) and Fe(III) states in nature and these forms are taken up selectively by different bacteria (List et al., 2019), being able to participate in the redox reactions as a medium and realizing the closure of iron cycle (Kappler et al., 2021). Metal-respiring bacteria can gain energy directly from electron transfer to Fe(III), and fermentative iron reducers can use Fe(III) as a sink for excess reducing equivalents as a form of enhanced fermentation (Lovley et al., 2004; Lehours et al., 2010; Dalla Vecchia et al., 2014). Besides, Fe(III) bound to siderophores can enhance the photolytic production of reactive iron species in the euphotic zone and thus influence iron availability in aquatic systems (Barbeau et al., 2001, 2003). Generally, Fe(III) is deficiently soluble at circumneutral pH. In contrast, Fe(II) is commonly more soluble and more bioavailable at circumneutral pH, which greatly affects the growth and metabolism of microorganisms, and more importantly, regulates microbial community structure and function (Yin and Wang, 2019; Wang et al., 2020). The turnover of biotic iron redox species at circumneutral pH is catalyzed by Fe(III)-reducing bacteria as well as microaerophilic, phototrophic, and nitrate-reducing Fe(II)-oxidizing bacteria (Ehrlich et al., 2015).

Iron is a limiting nutrient for primary production (Hutchins and Bruland, 1998) and is in a low content in the marine euphotic zone (Yang et al., 2021), which affects the growth, metabolism and function of microbes, and then further alters the structure and diversity of microbial community (Toulza et al., 2012; Liu et al., 2017). In recent years, a deal diversity of microorganisms that acquire energy from iron redox transformations has emerged, and researches on their physiology, ecology and environmental effect are growing. Nevertheless, elucidating the individual contribution of certain biotic or abiotic processes during iron cycling is extremely challenging (Kappler and Bryce, 2017). In our recent researches, we found that the suspended particles in the marine euphotic zone contained a large number of semiconducting iron minerals with remarkable photoelectric response, such as goethite, hematite, magnetite, and pyrite (Lu et al., 2019; Liu J. et al., 2020; Supplementary Figure 1 and Supplementary Table 1). Especially, as one of hydrated iron oxides, goethite has been playing an important role in photochemical cycling of iron, including photocatalytic oxidation (Wells et al., 1991; Jung et al., 2021) and photoreduction process (Pehkonen et al., 1993, 1995). There were limited studies focusing on the effect of goethite photoreduction on electron-transfer reactions within atmospheric water droplets (Faust, 1994) and its role in the surface water of the acidic conditions (Ercilla et al., 2009). However, the phenomenon of photoreduction of semiconducting iron minerals in the weekly alkaline marine environment has been neglected. Moreover, there are scarcely any researches on the function of dissolved Fe(II) from the photoreduction of goethite on native microbial community in the marine euphotic zone.

Correspondingly, based on our previous work, we selected typical mineral goethite to construct photoreduction systems and mineral–microorganism systems to probe into the impact of ferrous ions on microbial community in the marine euphotic zone. Experimental researches were conducted in view of the semiconducting properties and photoreduction of goethite in simulated seawater, as well as the regulation of dissolved Fe(II) on the structure and diversity of microbial community. The targets of this research were as follows: (a) analyzing the mineral phase and the morphology characterization of goethite electrodes through energy disperse spectroscopy (EDS), Raman, X-ray diffraction (XRD), environmental scanning electron microscope (ESEM), and atomic force microscope (AFM), and elucidating its semiconducting properties by ultraviolet–visible (UV-Vis) and electrochemical measurements; (b) investigating the content of dissolved Fe(II) from photoreduction of goethite under light conditions in simulated seawater; (c) exploring the effect of dissolved Fe(II) on structure and diversity of microbial community. All these might help highlight a phenomenon that photoreduction of semiconducting mineral goethite may occur in the marine euphotic zone. And this research has made attempts to reveal the effect of goethite photoreduction on the microbial community, which would broaden the comprehension of energy flow and biogeochemical carbon cycle. The source of ferrous ions and its availability to microbial community in the critical zone could be much greater than previously thought.

## Materials and Methods

### Samples Collection and Preparation of Artificial Seawater

#### Samples Collection and Processing

Samples of natural seawater were collected from the offshore area and faraway shore of the marine euphotic zone of the Yellow Sea, China, located at 119°28′E, 34°46′N with a depth of 5 m. The pH was 7.74 and 7.72 of the offshore area and faraway shore, respectively. The distance was about 0.2 km of the nearshore and 5 km of the faraway shore. The initial nearshore samples and faraway shore samples were marked “Offshore” and “Faraway,” respectively, and both of them were, respectively, divided into three groups to carry out subsequent experiments. To assure the sterile conditions, samples for microorganism cultivation were collected through sterile fillings and stored at 4°C as sources of inocula for further microbiological experiments.

#### Preparation of Artificial Simulated Seawater

The artificial simulated seawater was prepared 1 L with the composition of NaCl (26.518 g⋅L^–1^), MgSO_4_ (3.305 g⋅L^–1^), MgCl_2_ (2.447 g⋅L^–1^), CaCl_2_ (1.141 g⋅L^–1^), KCl (0.725 g⋅L^–1^), NaHCO_3_ (0.202 g⋅L^–1^) and NaBr (0.083 g⋅L^–1^), and pH was 8.20 (according to the data from Third Institute of Oceanography, Ministry of Natural Resources, China). According to the value of total organic carbon (TOC) of the natural seawater (2–10 mg⋅L^–1^) (Liu et al., 2021), 10 mg⋅L^–1^ was selected as the standard. Meanwhile, 40.45 mL 0.5 g⋅L^–1^ fulvic acid (OKA Biotechnology, Beijing, China) was applied as the hole trapping agent based on the TOC value of fulvic acid to achieve the content of simulated seawater TOC. The well-prepared artificial seawater was set in glovebox to avoid influence of oxygen in air and was used for the subsequent experiments of goethite photoreduction.

### Preparation of Goethite

#### Preparation of Synthesized Goethite Powder

Initially, adding 180 mL 5 mol⋅L^–1^ NaOH into 100 mL 1 mol⋅L^–1^ Fe(NO_3_)⋅9H_2_O solution and diluted to 2 L by Milli-Q deionized water (specific resistance ≥18.2 MΩ⋅cm^–1^). Then, the solution was mixed evenly and heated at 70°C for 60 h in a polypropylene bottle. After that, goethite was synthesized after centrifuging and cleaning for at least three times with Milli-Q deionized water (Schwertmann and Cornell, 2000).

#### Preparation of Synthesized Goethite Electrodes

Goethite electrodes were synthesized in bulk by coating method. At first, accurately weighing grinded 0.08 g synthesized goethite powder. Adding 3 mL anhydrous ethanol to dilute and carrying on ultrasonic dispersion for 5 min. Next, adding 150 μL Nafion emulsion into the mixture (anhydrous ethanol: Nafion = 20: 1) and ultrasonic dispersion for 5 min evenly again. Afterward, the mixture stirred well was dropped by 1 mL pipette on the conductive surface of fluorine doped tin oxide (FTO) (TCE15, Pilkington, Japan) electrode with an effective area of 4.0 cm × 2.5 cm. Then, a layer film of mineral powder was attached to the surface of FTO after drying naturally. Before using the FTO electrode, it was ultrasonically cleaned with acetone, anhydrous ethanol, and deionized water separately for 30 min. Three synthesized goethite electrodes (Goethite1, Goethite2, and Goethite3) among the batch were selected for subsequent tests to illustrate the parallelism, repeatability, and reasonability of experiments. All of the reagents used in this paper were analytical reagents.

### Mineralogical Characterization of Goethite

X-ray diffraction was performed by using an X-ray diffractometer (Rigaku Dmax-2400, Japan) equipped with Cu Kα irradiation (λ = 1.5406 Å). The patterns were recorded from 10° to 80° (2θ) with a scanning speed of 4°/min at 40 kV and 40 mA.

Mineral phase analysis of synthesized goethite was measured by Raman spectra (Confocal Raman Micro-spectrometer, Renishaw inVia Reflex, United Kingdom) equipped with a 532 nm laser. The laser intensity was 0.5% to avoid damage of samples and the diameter of the focus spot was 1 μm approximately with a long working distance 50× objective. The scanning range was 100–1000 cm^–1^ with 1 cm^–1^ of spectral resolution.

The particle size and height of goethite electrodes were measured by AFM (Bruker Dimension ICON, United States) with the probe of OTESPA-R3 (f_0_: 300 kHz, k: 26 N/m) to obtain the morphology image in the Tapping Mode. NanoScope Analysis software was used to measure the morphology of electrode surface.

The micro morphology of synthesized goethite powder was observed by ESEM (Thermal Fisher Quattro S, United States) operated at 15.00 kV under the secondary electron image mode. The chemical element compositions were detected by EDS equipped with ESEM.

The optical band gap of goethite electrodes was determined by UV-Vis diffuse reflection spectra (DRS) collected by UV-Vis absorption spectrophotometer (UV 3600 Plus, Japan). The integrating sphere was from 350 to 700 nm and the slit width was 3.0 nm. All the spectra were subtracted with the baseline of the bare FTO substrate, and BaSO_4_ was used as reference.

### Semiconducting and Electrochemical Measurements of Goethite

The conventional three-electrode configuration system in a quartz cylinder cell was adopted to carry out the cyclic voltammetry (CV), Mott–Schottky plots and real-time amperometric measurements (I-t). It consisted of the goethite electrodes for the working electrodes, a 213-type platinum electrode for the auxiliary electrode and a 232-type saturated calomel electrode (SCE) for the reference electrode.

Cyclic voltammetry was used to evaluate the properties of mineral electrodes and determine the potential parameters of redox reaction (Almeida and Giannetti, 2003). Reduction reaction of electrodes occurred when scanning to the negative potential, and the oxidation reaction occurred when scanning to the positive potential, which oxidized the previous reduction products. The reaction was more likely to occur with higher current of the oxidation/reduction peaks. Measurement conditions of CV curves were conducted in 0.1 M Na_2_SO_4_ from the open circuit potential as the initial potential, within the potential from −1 to 1 V at the scanning speed of 0.05 V/s. The scan segments were six. The fifth and sixth segments with stable results were selected as CV diagrams. Mott–Schottky plots were used to obtain the conductivity properties, flat band potential and carrier concentration of goethite. They were also performed in 0.1 M Na_2_SO_4_ within the potential window from −0.5 to −0.1 V with the frequency of 100 Hz. Dark/light I-t curves were measured in electrolyte of 0.1 M Na_2_SO_4_ with 1.0 M anhydrous ethanol (hole trapping agent) at a constant potential of 1.2 V within 420 s at the interval of 0.1 s to reflect the intensity and process of photocurrent response of goethite. The photocurrent response curves of goethite were obtained by periodically applying light and dark conditions in 60 s. Circular light and dark conditions were achieved by an external light-emitting diode (LED) with a working wavelength from 400 to 700 nm. The luminous flus were 1210 lx (within the normal range of nature) in backlighting method, which was measured by the TES-1332A photosynthetic radiometer (TES Electrical Electronic Corp., Taiwan). All of these electrochemical measurements of goethite were determined by an electrochemical workstation (CHI 760E Shanghai Chenhua Instrument, Shanghai, China). All potentials were referenced to the SCE (0.245 V vs. SHE) unless otherwise stated in whole paper.

The goethite band gap was calculated according to Tauc curve Equation 1 (Pankove, 1971):


(1) $$\alpha \,h\,\upsilon ={\text{A}}_{0}\,{\left(h\,\upsilon -E_{g}\right)}^{\text{m}}$$

In this equation, α is the absorption coefficient, *h*υ is the photon energy (eV), *E*_*g*_ is band gap energy (eV), A_0_ and m are both constants. For the direct electron transition, m = 0.5, and for the indirect electron transition, m = 2.

Based on the analysis of the band gap, impedance-potential was carried out. The information about the flat-band potential and carrier concentration of the goethite film was calculated and analyzed by the Mott–Schottky (Sukhotin et al., 1989). The Mott–Schottky equation relates *C* (the capacitance of space charge layer, F⋅cm^2^) to the *N*_*q*_ (carrier concentration, cm^–3^) as shown in the following Equation 2:


(2) $$C^{-2}=2\,\left(E-E_{f\,b}-k\,T/q\right)/\varepsilon \,\varepsilon_{0}\,q\,A^{2}\,N_{q}$$

In this equation, *E* is the electrode potential (V), *E*_*fb*_ is the flat band potential (V), *k* is Boltzmann constant, *T* is temperature (K), *q* is fundamental charge constant, ε is dielectric constant (F/m), ε*_0_* is vacuum permittivity (F/m) and *A* is the electrode area (cm^2^). Wherein, the dielectric constant of goethite is 25–50 F/cm. At room temperature, *kT*/*q* is about 25 mV, which can be negligible.

### Reaction Configuration of Photoreduction System and Determination of Dissolved Fe(II)

The photoreduction systems were conducted in anaerobic culture jars. Light conditions were achieved by an external LED with the wavelength of 400–700 nm and the luminous flux of 1585 lx (within the normal range of nature). Dark conditions were created by a cylindrical opaque cover. The experimental systems were composed of four devices, “Goe + Light,” “Goe + Dark,” “No Goe + Light,” and “No Goe + Dark” with simulated seawater. Each of them was 100 mL and equipped with stirring magneton at the speed of 400 rpm. The amount of goethite added to the devices was 0.4 g⋅L^–1^. Samples were taken every 24 h and the volume of each sample was 1 mL through 0.22 μm filter membrane each time.

The concentration of dissolved Fe(II) was measured by ferrozine mono-sodium salt. The absorbance under the optical density at 562 nm (OD_562_) was determined by another UV-Vis absorption spectrophotometer (Thermo Fisher Evolution 220, United States). The concentration of dissolved Fe(II) was obtained according to the standard curve of absorbance–concentration.

### Construction of Goethite-Microorganism and Fe^2+^-Microorganism Systems

Goethite-microorganism and Fe^2+^-microorganism systems were constructed to explore the effect and regulation of ferrous ions on the microbial community. The final concentration of experimental devices was 0.4 g⋅L^–1^ with goethite and 0.05 mol⋅L^–1^ with FeCl_2_4H_2_O (Fe^2+^), respectively. The experimental groups were divided into “euphotic seawater-goethite,” “euphotic seawater-Fe^2+^,” and “euphotic seawater-blank.” All devices were cultured in a shaker incubator at constant temperature of 30°C with the speed of 150 rpm. All systems were monitored by detecting the concentration of dissolved Fe(II) which was also measured through the assay of ferrozine mono-sodium salt under the optical density at 562 nm (OD_562_). In the meanwhile, the concentration of dissolved Fe(II) was obtained according to the standard curve of absorbance–concentration. Samples were taken on the 15th and 30th days for high-throughput sequencing analysis of microbial community.

### Analysis of Structure, Diversity, and Functional Prediction of Microbial Community

Microbial community of marine samples were taken in the 16S rRNA gene sequence reads. In view of the microbial community structure, the samples were analyzed at the V3/V4 regions of 16S rRNA gene from the perspectives of phylum, class, order, family, and genus. The V3/V4 hypervariable regions of bacterial 16S rRNA gene were amplified with the primers 357 F and 806 R (Huber et al., 2007). Majorbio Company performed the high-throughput paired-end Illumina MiSeq sequencing, and high-grade reads were obtained after complex processing (De Mandal et al., 2016). DNA was extracted by FastDNA^®^ Spin Kit (MP Biomedicals, United States). In sequencing experiments, firstly, the genomic DNA was extracted and monitored by 1% agarose gel electrophoresis. Then, PCR amplification was performed, and specific primers with barcode were synthesized by the designated sequencing area. According to the quantitative results of electrophoresis, PCR products were detected and quantified by fluorescence. After that, the MiSeq library was constructed. Finally, MiSeq sequencing was performed, and the sequence of template DNA fragment could be obtained by counting the fluorescence signal results (Majorbio Company, Shanghai, China). Optimal sequence clustering was performed as operational classification units operational taxonomic units (OTUs) for species classification at 97% similarity level, counting the relative richness information of each OUT in samples. Alpha diversity, including Simpson, Shannon, and Chao 1 indexes, was used to analyze microbial community diversity and abundance of microbial community in each system. Additionally, the heatmap which can reflect the correlation was applied for displaying the changes in the richness of different species through the color gradient of each color patch. The red color represents the higher positive correlation. The blue color represents the higher negative correlation.

## Results and Discussion

### Mineralogical Characterization of Goethite

#### Mineral Phase of Goethite

**FIGURE 1:**
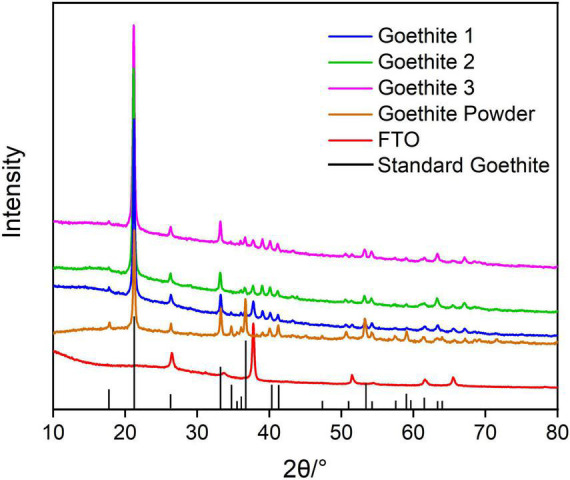
X-ray diffraction patterns of synthetic goethite.

The synthetic iron mineral analyzed by XRD was corresponding to the characteristic diffraction crystal planes of standard goethite (ICDD: 01-081-0464). Therefore, XRD analysis showed that the brown–yellow synthetic semiconducting mineral was high purity goethite (Figure 1). Raman also further confirmed the purity of synthetic goethite (Supplementary Figure 2).

#### Morphology and Structure of Goethite

**FIGURE 2:**
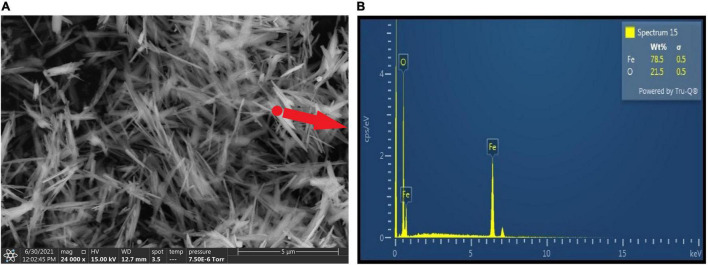
Environmental scanning electron microscope images **(A)** and EDS **(B)** of synthetic goethite.

Initially, the surface morphological characterization of the synthetic goethite was observed under ESEM (Figure 2A). The synthesized goethite showed acicular structure with high crystallization degree at secondary electron image with 24,000× magnification. And the particle was uniformly distributed without obvious agglomeration. Each acicular particle was approximately 3 μm in length. In addition, the EDS data suggested the dominant elements were Fe and O (Figure 2B), indicating again high purity of synthesized goethite.

Ulteriorly, the thickness, 2D and 3D morphology of goethite electrodes were measured by AFM. At *z*-axis, the average height difference of goethite was measured between the high and low steps, indicating its thickness was approximately 4.5 μm. The thickness of goethite electrodes was appropriate for light transmission in subsequent electrochemical measurements (Supplementary Figures 3, 4).

### Semiconducting Properties for Band Gap and Flat Band Potential of Goethite

**FIGURE 3:**
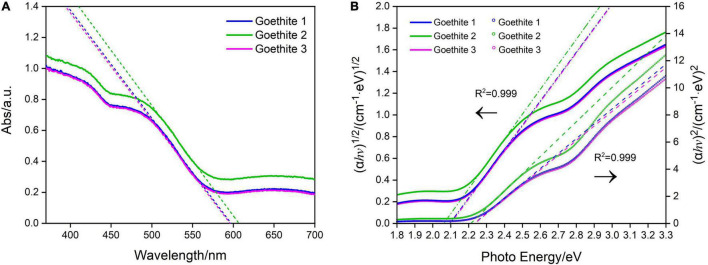
Ultraviolet–visible absorption spectra **(A)** and Tauc plots of synthetic goethite electrodes for the direct and indirect band gaps **(B)**.

The band gap refers to the difference of energy band between the top of the valence band and the bottom of the conduction band, which is the major factor to determine the semiconducting properties (Grätzel, 2001). The band gap of goethite electrodes in this research was investigated *via* UV-Vis absorption spectroscopy, exhibiting the typical absorption edge of 600 nm (Figure 3A). The strong absorption within visible light region at 400–600 nm was generated by the excitation of 3d–3d orbital electron spin barrier of Fe^3+^ (Debnath and Anderson, 1982).

The indirect and direct band gap of goethite were 2.08 and 2.23 eV, respectively, as calculated by Tauc Equation 1 (Tauc, 1966; Figure 3B). Based on the direct band gap value of 2.23 eV, goethite enabled easy electrons excitation and transition from valence band to conduction band. The active electron-hole pairs can be generated in the lattice, thus promoting a series of redox reactions.

**FIGURE 4:**
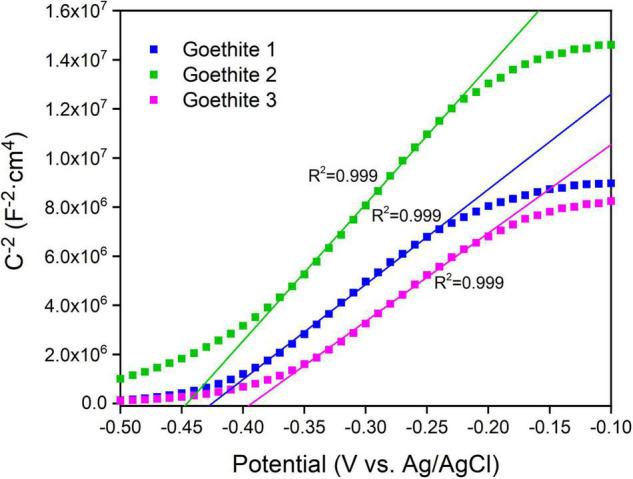
Mott–Schottky plots of synthetic goethite electrodes measured at 100 Hz.

Except for the band gap, the flat band potential (*E*_*fb*_) is another vital parameter to characterize the energy band structure of photocatalytic semiconductors in solution system. The slopes of Mott–Schottky curves (Figure 4) were positive, indicating that goethite is an *n*-type semiconductor. According to the fitting line, the *E*_*fb*_ of goethite was about −0.40 to −0.45 V, suggesting a relatively low electron-hole binding rate, which was favorable for the surface catalytic reaction of goethite. By substituting the slope of the fitting line into Equation 2, the carrier concentration of goethite was calculated as 4.45 × 10^19^ cm^–3^ in the 0.1 M Na_2_SO_4_ solution. The higher the carrier concentration was, the greater the current would be generated. Therefore, goethite had an excellent surface catalytic activity and electron transfer efficiency, which possibly facilitated electrochemical reduction reactions under excitation conditions.

### Properties for Photo-Response, Electron Transfer, and Redox Activity of Goethite

The electrochemical characteristics of goethite were further investigated, including CV and real-time amperometric measurements (I-t), to evaluate the electrochemical properties and redox potential of goethite electrodes.

On the side of positive bias potential, the curves were almost horizontal straight lines. The current value was close to zero and remained stable with the increase of potential. The results also demonstrated that no photocarriers were generated and that it was difficult for electron transfer and chemical reactions to occur, resulting in a small current/potential ratio. Correspondingly, a significant current change produced on the side of negative bias potential due to the reduction–oxidation cycle of the variable metal iron in goethite.

**FIGURE 5:**
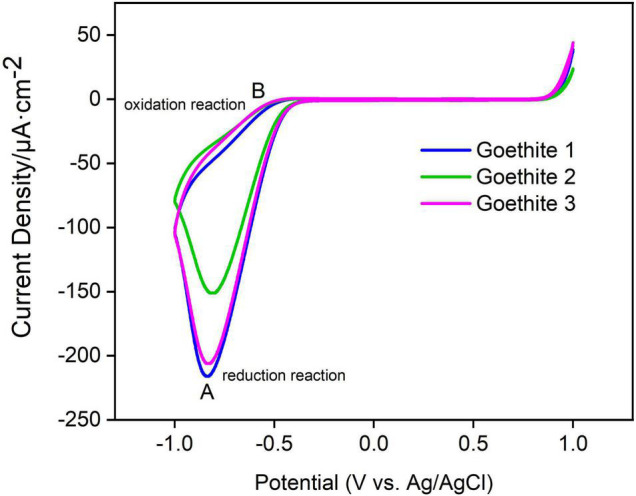
Cyclic voltammetry curves of synthetic goethite electrodes.

In Figures 5A,B represented reduction and oxidation peaks of goethite, respectively. The A and B peaks were a pair of symmetric peaks, and the reduction reaction at A was Equation 3:


(3) $$\alpha -\text{F}\,\text{e}\,\text{O}\,\text{O}\,\text{H}+3\,H^{+}+{\text{e}}^{-}\to {\text{Fe}}^{2+}+2\,H_{2}\,\text{O},-0.83\,V$$

The oxidation reaction at B was Equation 4:


(4) $${\text{Fe}}^{2+}\to {\text{Fe}}^{3+}+{\text{e}}^{-},-0.51\,V$$

In addition, the peak positions and peak heights of A and B among three goethite electrodes presented the same trend, which confirmed that the electron transfer of goethite could occur under light. The photoelectron transfer activity of goethite was further measured in order to better characterize the ability of goethite electrodes to generate electron-hole pairs under visible light.

**FIGURE 6:**
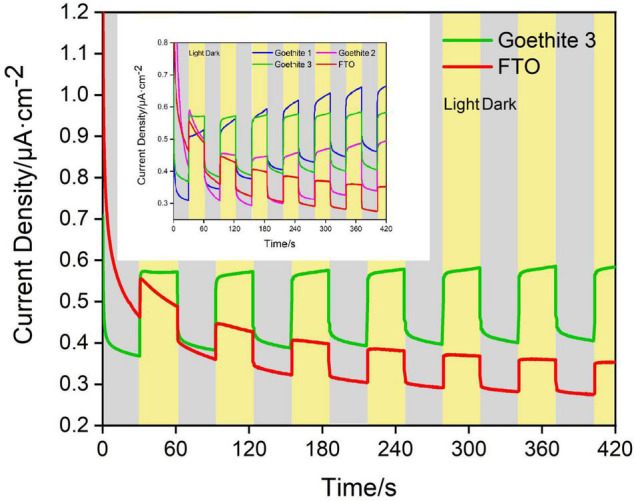
Dark/light amperometric I-t curves for synthetic goethite electrodes with 1 M alcohol.

Based on CV analysis of electrode reactivity, real-time amperometric measurements (I-t) were carried out in an actual reaction system to identify the in-depth influence of light/dark conditions on electrode electrochemistry and photo-response of goethite. Under the dark condition, it was difficult for goethite electrodes to transfer electrons due to the low activity, resulting in a low current with weak response. On the contrary, goethite was capable of receiving light to generate photocarriers, which promoted the electron transfer and surface chemical reaction. The photocurrent and dark current density of one of three goethite electrodes were analyzed. The test of I-t under 1.2 V indicated that (Figure 6 and Table 1): the increment of the average photocurrent density (0.575 μA/cm^2^) relative to the dark current density (0.399 μA/cm^2^) was about 0.176 μA/cm^2^, with an increment ratio of about 44.11%. When the light was shut off, the photocurrent fell back to the baseline in short order. Compared with the photocurrent density of the blank electrode FTO (0.404 μA/cm^2^), the average photocurrent density of the goethite electrode (0.575 μA/cm^2^) was about 1.5 times. In addition, the increment of goethite electrode was about two times better than that of the FTO. The photoelectron-hole separation occurred in goethite under light, and the photocarriers greatly enhanced the electron transfer, emerging prominent photocurrent response in the system. By this token, it further demonstrated that goethite can be activated by light and appeared advantageous semiconducting properties of photoelectrochemical response under light irradiation.

**TABLE 1:** Current density of goethite electrode under light and dark (voltage: 1.2 V).

Potential 1.2 V	Photocurrent density/ (μA/cm^2^)	Dark current density/ (μA/cm^2^)	Increment/ (μA/cm^2^)	Increment ratio/%
Goethite electrode	0.575	0.399	0.176	44.11
Blank (FTO)	0.404	0.312	0.092	29.49

### Photoreduction Characteristic of Goethite in Simulated Seawater

**FIGURE 7:**
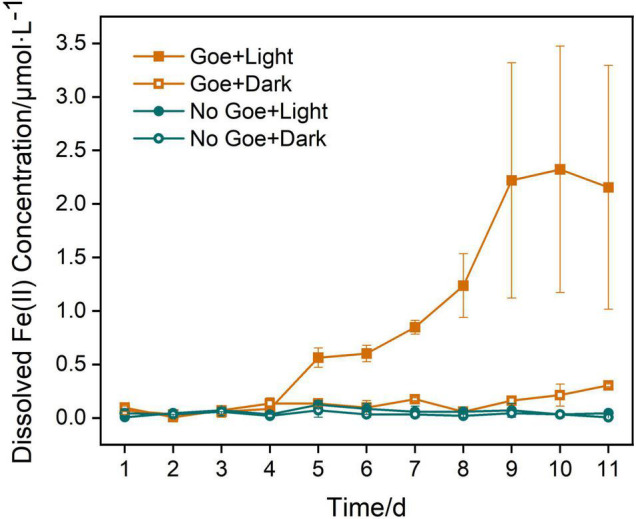
Concentration of Fe(II) dissolved by photoreduction of goethite.

Goethite was applied to establish simulation systems of the marine euphotic zone to analyze Fe(II) dissolved by photoreduction in the lab. Photoreduction of goethite were carried out in parallel experiments under different conditions of “Goe + Light,” “Goe + Dark,” “No Goe + Light,” and “No Goe + Dark” during 11 days. The results demonstrated that photoelectrons of goethite can be generated by photoreduction in the simulated artificial seawater (pH = 7.9–8.2) under light. The dissolution rate and concentration of dissolved Fe(II) from goethite stimulated by light were significantly higher than that in dark groups within 11 days (Figure 7). Specifically, in the initial 4 days, on account of the unstable state of systems, the photoreduction rate of goethite was slow. The content of dissolved Fe(II) fluctuated under different conditions, which may be caused by detection deviation. From the 4th day of reaction, the figure for the “Goe + Light” group began to arise a distinct increase. On the 8th and 9th days, the largest growth rate of reaction appeared. Then the content of dissolved Fe(II) continued to increase and reached the peak value of an experimental cycle on the 10th day. On the other hand, in the “Goe + Dark” group, the dissolution rate of Fe^2+^ was slow and began to rise slightly from the 9th day. While in the groups of “No Goe + Light” and “No Goe + Dark,” there was almost no dissolution of Fe^2+^. On the 10th day, the dissolved Fe(II) was 2.32 μM with light, which was nearly 11 times than that of “Goe + Dark” group (0.21 μM). And on the 11th day, the concentration of dissolved Fe(II) was still only 0.30 μM in control groups without light. Relatively, the dissolved Fe(II) was 2.15 μM with light in the group of “Goe + Light,” which was 616.67% higher than that of the “Goe + Dark” group. Although the content of dissolved Fe(II) was slightly reduced from the 11th day in “Goe + Light” group, it may be caused by the oxidation of Fe^2+^ to Fe^3+^, indicating that the systems were in the dynamic equilibrium state of Fe^2+^–Fe^3+^ transformation. Besides, due to the limitations of laboratory simulation, the minimum amount of Fe^2+^ dissolved by photoreduction was at least like the average dissolution rate of Fe(II) in our experiments, i.e., 0.54 μM/day. That is, the amount and rate of dissolved Fe(II) achieved by photoreduction of iron minerals in natural euphotic zone system will beyond this level. In general, this experiment indicated that Fe^2+^ can be dissolved by photoreduction of goethite in the weekly alkaline environment of the marine euphotic zone under light. This photoreduction reaction is an important source of iron in the marine environment. The photoreduction of semiconducting iron minerals can dissolve Fe(II), which may have an impact on the native microbial community and energy flow mediated by semiconducting iron minerals.

### Effect of Ferrous Ions on Microbial Community

To further explore the effect of Fe(II) dissolved by goethite photoreduction on the microbial community, seawater samples were collected from the marine euphotic zone of the Yellow Sea. Herein, the initial nearshore samples and samples from faraway shore were marked “Offshore” and “Faraway,” respectively. The systems of “euphotic seawater-goethite” (Offshore1, Faraway1), “euphotic seawater-Fe^2+^” (Offshore2, Faraway2) and “euphotic seawater-blank” (Offshore3, Faraway3) were established using the original euphotic zone community with three groups of parallel experiments for each condition. And a representative group of data was selected to draw diagrams and tables. Among them, 0.4 g/L goethite was added to the system of “euphotic seawater-goethite,” and 5 mM Fe^2+^ was added to the system of “euphotic seawater-Fe^2+^.” Each system reaction was cultured lasted for 30 days, and community sequencing analysis was performed at the beginning, on the 15th and 30th days, respectively.

#### Alpha Diversity Analysis

**FIGURE 8:**
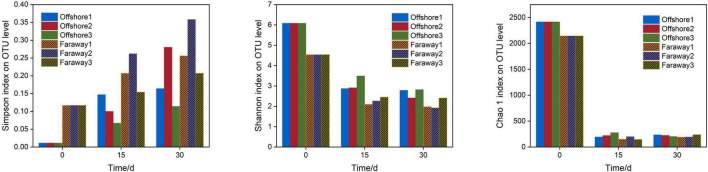
Bar charts for alpha diversity, including Simpson, Shannon, and Chao 1 indexes of community on the initial day, 15 and 30 days (Offshore represents the distance of 0.2 km from shore. Faraway represents the distance of 5 km from shore).

Alpha diversity analysis (Figure 8) demonstrated that Simpson index have been continuously increasing with the growth of culture time for both offshore and far-shore samples (from 0.011–0.117 to 0.114–0.358). On the contrary, Shannon index decreased continuously with the growth of culture time (from 4.537–6.080 to 1.924–2.819). From the two relative indexes, Shannon and Simpson, it can be concluded that the community diversity decreased gradually with increasing culture time for both nearshore and far-shore samples. For another, the diversity of the far-shore samples was generally lower than that of the nearshore samples, whether in the beginning or after culture. On the other hand, Chao 1 index displayed the richness of samples from offshore and far-shore both decreased distinctly after cultivation, manifesting a dramatic reduction of microbial community richness. At the beginning, the richness of nearshore samples was slightly higher than that of far-shore samples. After 30 days’ cultivation, the richness levels of communities were roughly equal, but all were much lower than the initial figures (Figure 8).

Hence, all of these analysis indexes illustrated that the diversity and richness of community decreased, and the selectivity and adaptability of dominant microbial genera were outstanding after dissolved Fe(II) addition culture. The dissolved Fe(II) significantly affected the structure of microbial community and may promote the selection and enrichment of microorganisms related to of iron element cycle and metabolism. This result was consistent with previous research (Yin and Wang, 2019; Sindhu et al., 2021) about the effect of ferrous ions on the metabolism and diversity of microbial community. In a word, it preliminarily revealed that dissolved Fe(II) from the photoreduction of goethite had an impact on the diversity and richness of microbial community in the marine euphotic zone.

#### Microbial Community Composition

**FIGURE 9:**
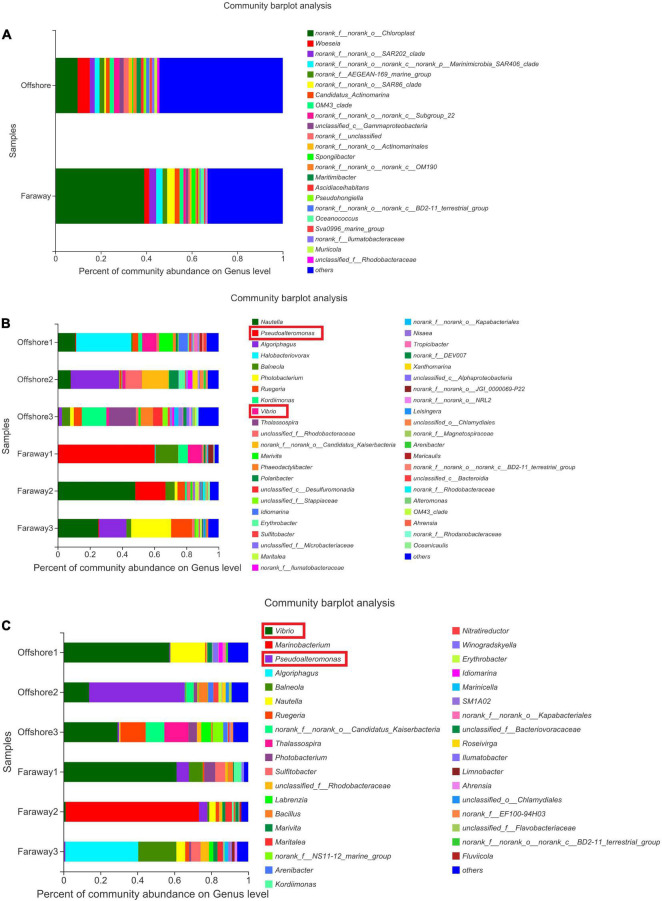
Microbial community structure of the initial day **(A)**, 15 days **(B)**, and 30 days **(C)** on genus level (Offshore represents the distance of 0.2 km from shore. Faraway represents the distance of 5 km from shore).

Notably, 16S rRNA sequencing on the community abundance and structure composition of microbial community indicated that the selectivity and adaptability of dominant microbial genera were enhanced after a long-term cultivation with dissolved Fe(II) addition. Specifically (Figure 9), at the beginning, the dominant genus was *Chloroplast*, which was widespread in seawater communities as primary productivity in marine (Figure 9A). After 15 days, the dominant genera were *Halobacteriovorax*, *Nautella*, *Algoriphagus*, and *Vibrio* in series samples of Offshore and *Pseudoalteromonas*, *Nautella*, *Algoriphagus*, and *Vibrio* in series samples of Faraway (Figure 9B). For 30 days’ cultivation, the structure of community changed and the advantages of *Vibrio* and *Pseudoalteromonas* were more highlighted in series samples of Offshore. *Vibrio*, *Marinobacterium*, and *Pseudoalteromonas* were more prominent in series samples of Faraway (Figure 9C). Therein, *Vibrio* was more prominent in Offshore1 and Faraway1 systems with goethite, *Pseudoalteromonas* was more prominent in Offshore2 and Faraway2 systems with Fe^2+^. Especially in Offshore2 system (with Fe^2+^), *Pseudoalteromonas* and *Vibrio* were accounting for 51.74 and 13.85% on the 30th day, respectively.

**FIGURE 10:**
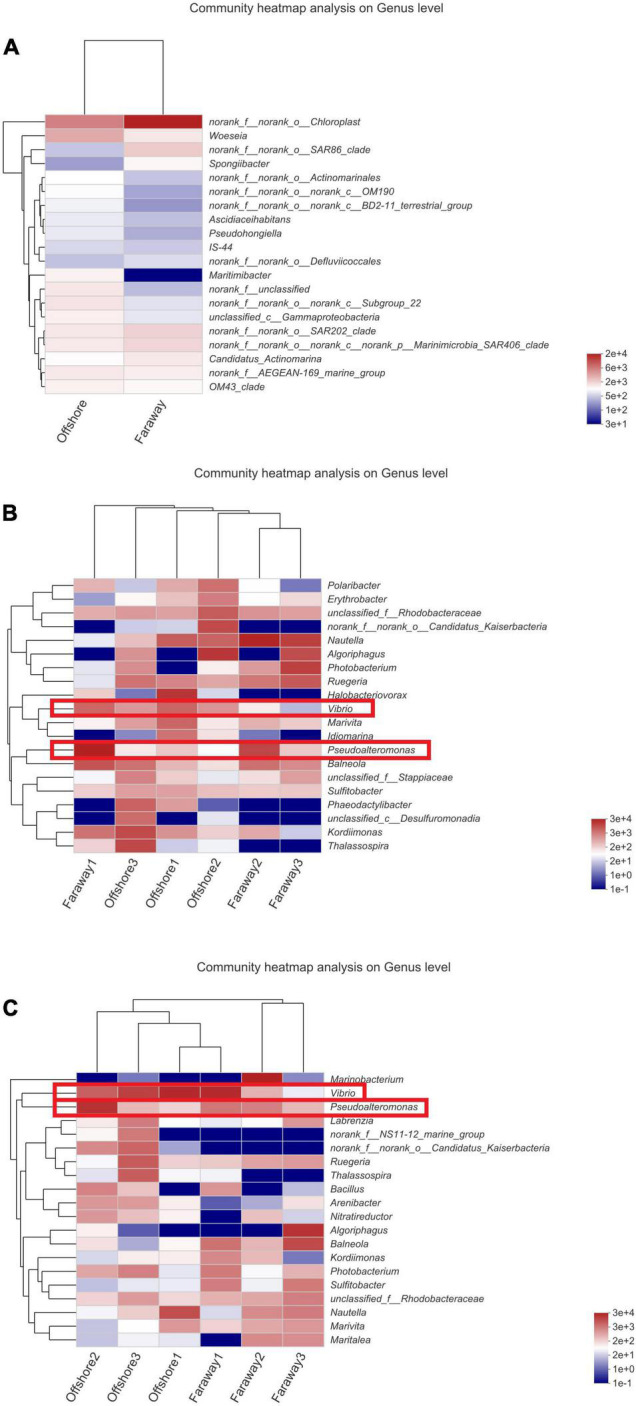
Heatmaps of the major taxa identified on the initial day **(A)**, 15 days **(B)**, and 30 days **(C)** (Offshore represents the distance of 0.2 km from shore. Faraway represents the distance of 5 km from shore).

Therefore, after 30 days, it can be assumed that two prominent strains were *Pseudoalteromonas* and *Vibrio*. In addition, the prominence of these two strains was more evident in heatmaps (Figure 10), which showed the distribution of the bacterial community throughout the experiments. In the heatmaps, the darker red color was corresponding to the higher correlation. The color of *Vibrio* in all systems reflected moderate correlation after 15 days (Figure 10B). After 30 days’ cultivation, the color tended toward red and the positive correlation increased (Figure 10C). The results indicated that the positive correlation of *Vibrio* promoted with the increase of culture time. It had the same tendency for *Pseudoalteromonas*, especially in Offshore2 (Figure 10C).

Thereby, the enrichment of bacteria suggested their noteworthy selectivity, adaptability, and superiority to the environment in which dissolved Fe(II) was added. The dominant bacteria identified in this paper have all been confirmed to be related to iron in recent researches (Table 2). In view of preponderant strains of *Vibrio*, *Pseudoalteromonas*, and *Marinobacterium* for 30 days, it was found that nutrients from Saharan dust in marine surface waters promoted the formation of *Vibrio* bloom, and dust-associated iron was a vital driving factor of *Vibrio* population dynamics (Westrich et al., 2016). Besides, iron was extremely scarce in the marine, but *Pseudoalteromonas* with broad iron storage capacity can produce a large number of TonB iron complex transporters for obtaining iron (Mazzotta et al., 2020). *Marinobacterium* species were commonly identified and were associated with metal corrosion processes, mainly in offshore oil structures (Cluff et al., 2014; Brauer et al., 2015). In a word, it preliminarily revealed that dissolved Fe(II) had an impact on the structure composition and diversity of microbial community. The photoreduction process of semiconducting iron minerals, such as goethite, indirectly affected and regulated the composition, diversity, richness, and the function of microbial community in the marine euphotic zone, which may also further regulate the related metabolic pathways of carbon cycle.

**TABLE 2:** Dominant microbial genera in alphabetical order and references associated with iron.

Genus	Function	References
*Marinobacterium*	Associated with metal corrosion processes, mainly in offshore oil structures.	Cluff et al., 2014; Brauer et al., 2015
*Nautella*	Having important roles in the initial formation of the structure of biofilms on surfaces.	Capão et al., 2020
*Pseudoalteromonas*	As potential marine Fe-oxidizing bacteria (FeOB), which can produce TonB iron complex transporters.	Ramírez et al., 2016; Mazzotta et al., 2020
*Vibrio*	Iron drives *Vibrio* population dynamics.	Westrich et al., 2016

## Conclusion

**FIGURE 11:**
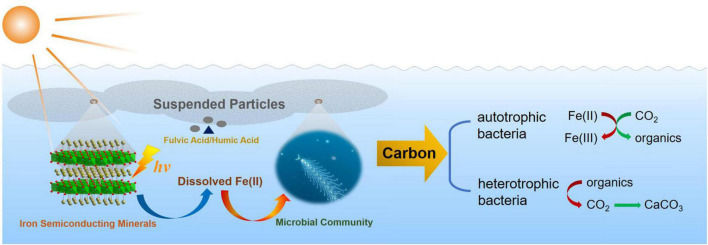
The diagram of effect and regulation of dissolved Fe(II) on microbial community and driving carbon cycle in the marine euphotic zone.

In summary, this work demonstrated the preponderant photo-response property of semiconducting goethite, which widely existed in the marine euphotic zone. Fe^2+^, deriving from the photoreduction dissolution of goethite, had a crucial impact on the structure, diversity, as well as the function of microbial community, which may also further regulate correlative metabolic pathways of carbon cycle in the marine euphotic zone. Furthermore, dissolved Fe(II) from the photoreduction of semiconducting iron minerals may affect and regulate the metabolic functions of autotrophic bacteria and the electron transfer of heterotrophic bacteria, as well as the changes of energy metabolism in the marine euphotic zone. There are two ways of carbon sequestration having been proposed, which will be conducted in the follow-up work. One is that the autotrophic microorganisms use the energy of Fe(II) to immobilize CO_2_ as organics in the euphotic zone. Another possible approach is that the heterotrophic microorganisms accelerate the conversion of CO_2_ into carbonate precipitation driven by energy cycle, and generate carbonate precipitation for carbon sequestration. That is because iron has an effect on the electron transfer process and metabolism of heterotrophic microorganisms, which promotes the use of organic substrates to generate CO_2_. Moreover, it will further reveal the changes of functional genes and transcriptomics in the euphotic microbial community. Generally speaking, the regulation level of dissolved Fe(II) on the euphotic microbial community will affect the electron transfer process, and the improvement of the oxidation capacity of organic substrates will influence the biogeochemical carbon balance and then drive carbon cycle in the euphotic zone (Figure 11). The photoreduction of goethite reported here advances our understanding that one of the vital sources of ferrous ions existed in the marine and how the photoreduction of semiconducting iron minerals indirectly affected the microbial community in the critical zone of the marine euphotic zone.

## Data Availability Statement

The original contributions presented in the study are included in the article/Supplementary Material, further inquiries can be directed to the corresponding authors.

## Author Contributions

JL and HD designed the experiments, analyzed the experimental results, and analyzed the data. JL carried out the experiments and wrote the manuscript. SY provided the experimental methods. JL, YS, and XG collected the marine samples. XG, YZL, and XJ assisted the tests. HD, XG, YZL, AL, and YL performed the revisions. AL funded the research. All authors agreed to submit the work to *Frontiers in Microbiology* and approved it for publication.

## Conflict of Interest

The authors declare that the research was conducted in the absence of any commercial or financial relationships that could be construed as a potential conflict of interest.

## Publisher’s Note

All claims expressed in this article are solely those of the authors and do not necessarily represent those of their affiliated organizations, or those of the publisher, the editors and the reviewers. Any product that may be evaluated in this article, or claim that may be made by its manufacturer, is not guaranteed or endorsed by the publisher.
