# Supplementary material for: Effect of Photoreduction of Semiconducting Iron Mineral—Goethite on Microbial Community in the Marine Euphotic Zone
Source: Front Microbiol. 2022 Apr 11;13:846441. doi: 10.3389/fmicb.2022.846441 (PMC9037543; doi:10.3389/fmicb.2022.846441)
Supplement: Supplementary file 1 [file Data_Sheet_1.docx]

## Supplementary Materials

### Composition of Typical Semiconducting Minerals in the Euphotic Zone

In our previous research (Liu et al., 2020), we have analyzed the composition of the suspended minerals in the marine euphotic zone of the Yellow Sea, China (Table 1). Suspended minerals contained semiconducting minerals of iron hydroxides and iron oxides, such as goethite and hematite, which retained prominent photoelectrochemical activity in response to the sunlight and photoelectric catalytic potential through electrochemical measurements.

The dried suspended particulate minerals were embedded with epoxy resin for the micromorphology observation under ESEM. The suspended minerals in ESEM secondary electron image were agglomerated and flocculent, and the typical mineral particles were acicular with a regular geometric shape (Figure 1a). The EDS data revealed that there was relatively high Fe element in minerals with geometric shapes and crystal morphology (Figure 1b), which was likely to Fe hydroxides or Fe oxides. Micro-Raman was applied to further explore the mineral composition and the mineral phase enriched in Fe element. As shown in the Raman spectra (Figure 1c), the iron mineral was goethite, which can be identified by four characteristic peaks 215 cm^-1^, 272 cm^-1^, 387 cm^-1^, 463 cm^-1^ (Thibeau et al., 1978; De Faria, 1997). Based on the results of EDS and Raman, we preliminarily concluded that Fe were enriched in euphotic zone, and the major iron mineral phase was goethite.

**Table 1.** Typical semiconducting minerals existed in marine euphotic zone (Liu et al., 2020).

| **Original Compositions in Marine Euphotic Zone** | **Semiconducting Minerals** |
| --- | --- |
|  | Goethite  Hematite  Anatase  Rutile  Brookite |
| **Photoreduction systems** | **Representative Iron Mineral** |
|  | Goethite |


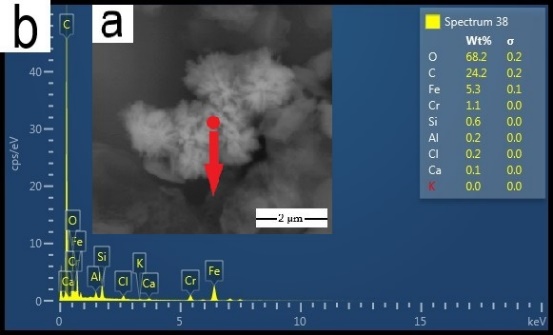

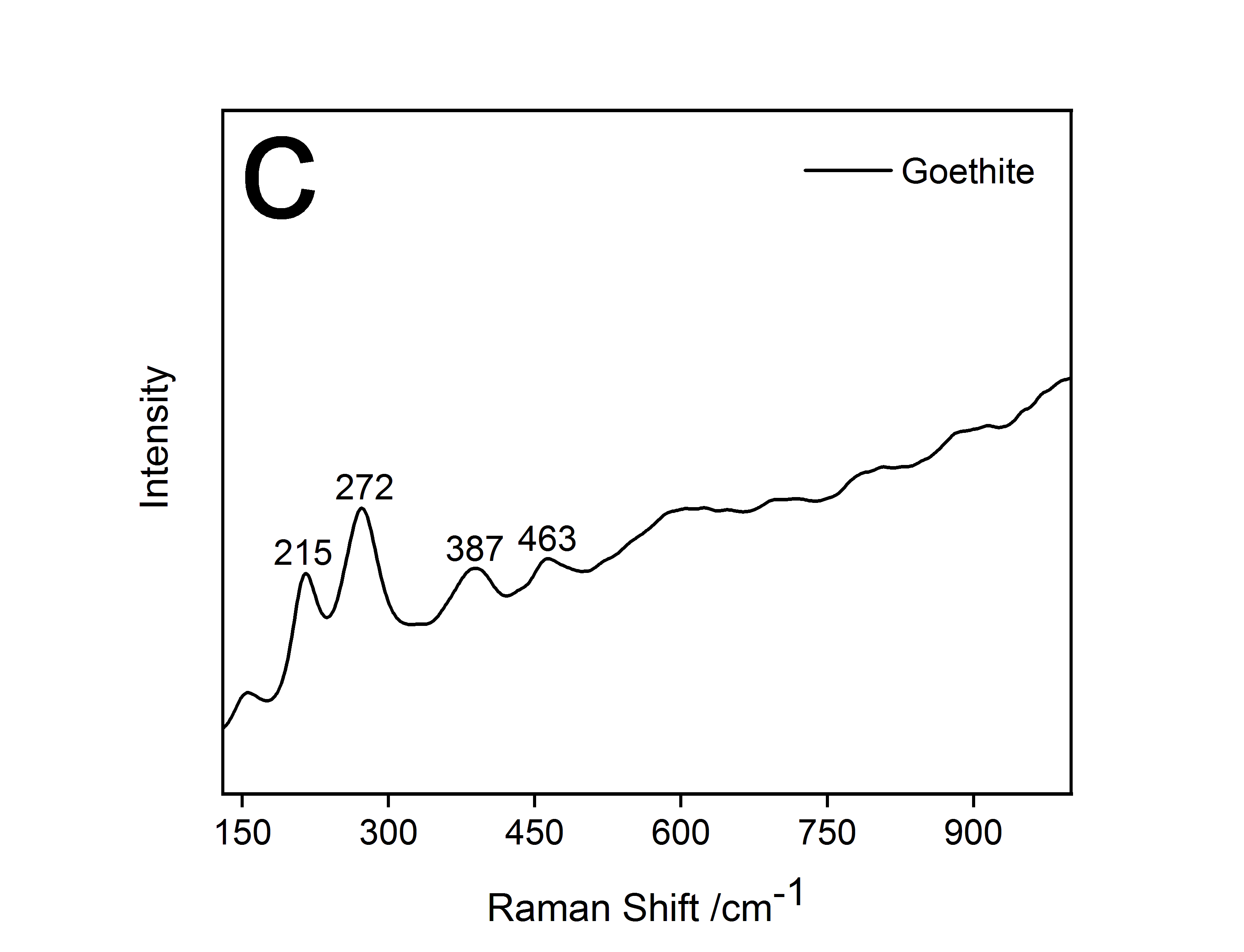


**Figure 1.** Morphology under ESEM (a), data of EDS (b) and Raman spectra (c) of goethite.

### Mineral Phase of Goethite

Raman was used to further confirm the synthetic mineral phase from micro scale (Figure 2). The Raman spectra of goethite showed obvious characteristic peaks at 300 cm^-1^, 387 cm^-1^, 479 cm^-1^, 549 cm^-1^ and 682 cm^-1^. Among, 300 cm^-1^ represented the symmetrical stretching vibration of Fe-OH. 387 cm^-1^ was the symmetric stretching vibration of Fe-O-Fe/-OH. 549 cm^-1^ characterized the asymmetric stretching vibration of Fe-OH and 682 cm^-1^ was the symmetric stretching vibration of Fe-O (De Faria, 1997; Oh et al., 1998). Therefore, the synthetic goethite can be used as purity minerals in the subsequent experiments.


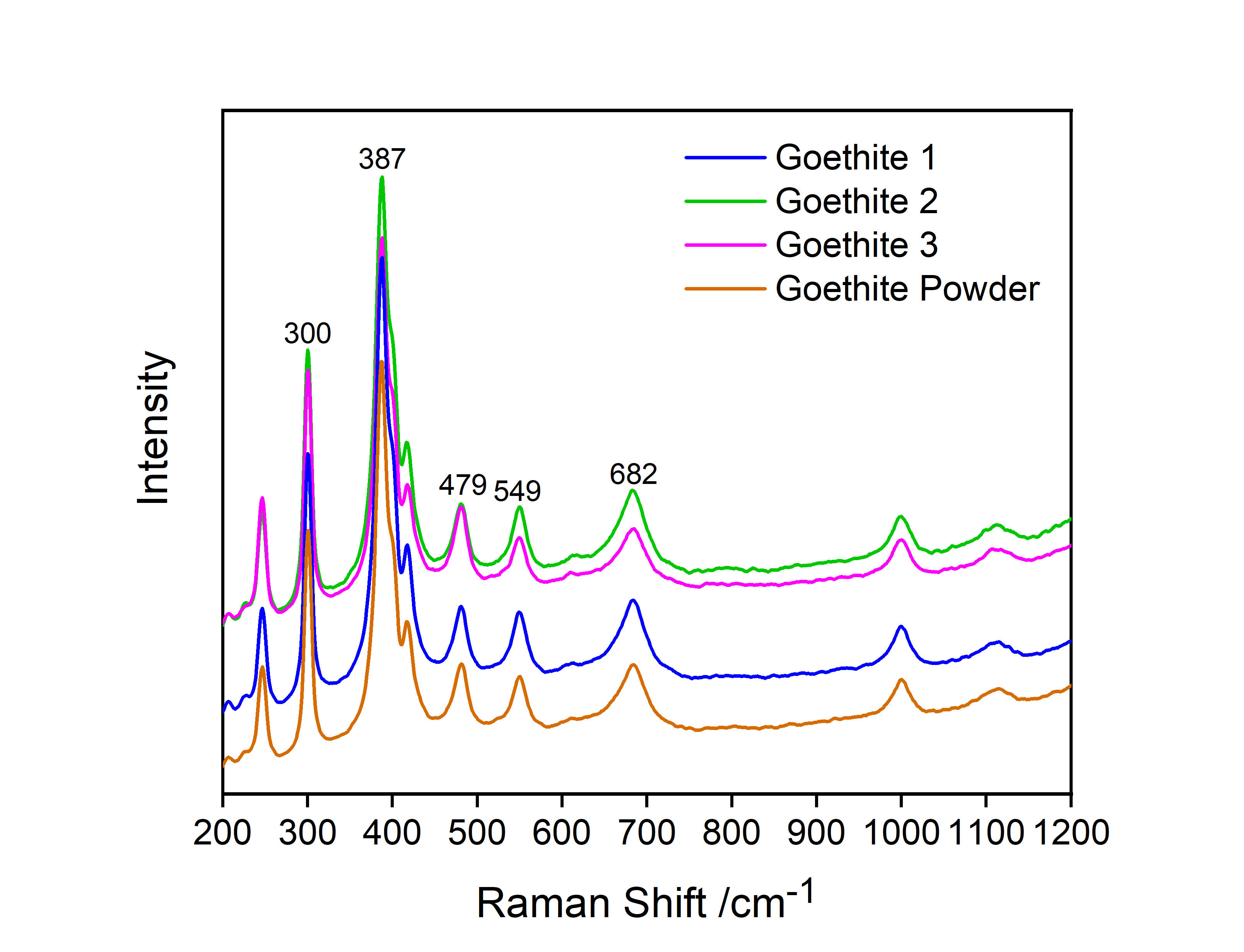


**Figure 2.** Raman patterns of synthetic goethite.

### Thickness of Synthetic Goethite Film

The thickness of goethite film was measured by AFM. At *z*-axis, the average height difference of goethite between the high and low steps was measured (Figure 3). Besides, the partial 2D (Figure 4a) and 3D (Figure 4b) morphology of synthetic goethite presented the tridimensional acicular and cluster structure with 2.5-3 µm size more intuitively.


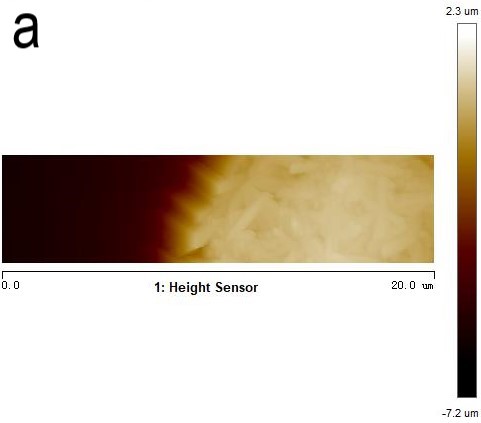

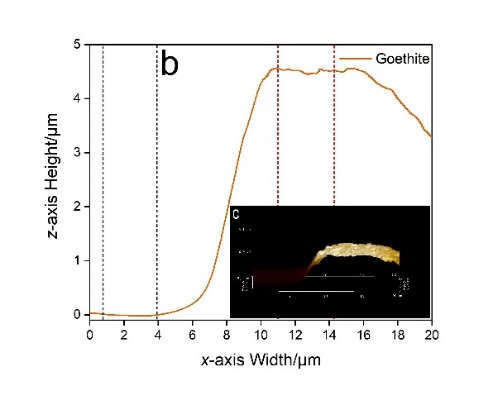


**Figure 3.** 2D of scan area (a), height of synthetic goethite (b) and 3D step under AFM (c).


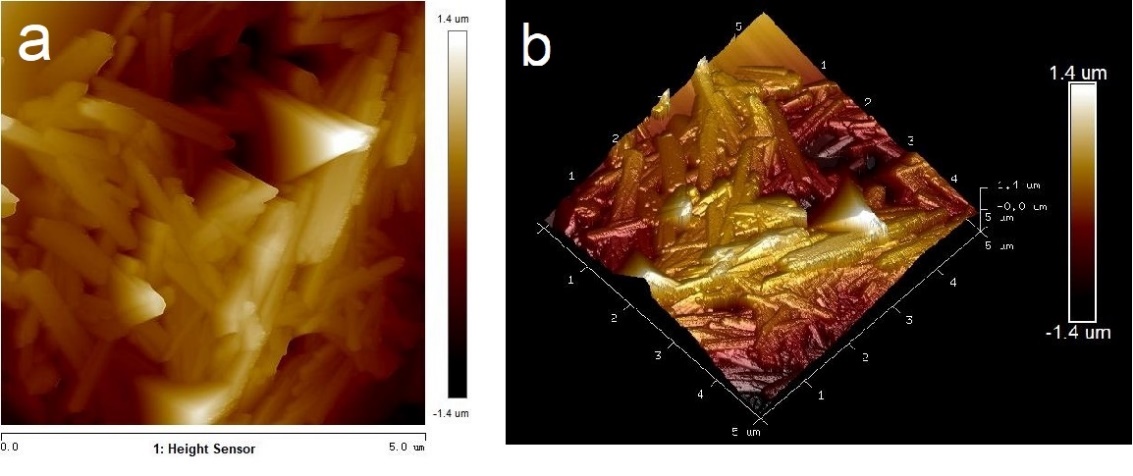


**Figure 4.** AFM 2D (a) and 3D (b) graphs of synthetic goethite.

### References

De Faria, D.L.A., Venâncio Silva, S., De Oliveira, M.T. (1997). Raman microspectroscopy of some iron oxides and oxyhydroxides. *J. Raman. Spectrosc.* 28(11), 873-878.

Liu, J., Sun, Y., Lu, A., Liu, Y., Ren, G., Li, Y., et al. (2020). Extracellular electron transfer of electrochemically active bacteria community promoted by semiconducting minerals with photo-response in marine euphotic zone. *Geomicrobiol. J.* 38(4), 329-339.

Oh, S.J., Cook, D.C., Townsend, H.E. (1998). Characterization of iron oxides commonly formed as corrosion products on steel. *Hyperfine Interact.* 112, 59-65.

Thibeau, R.J., Brown, C.W., Heidersbach, R.H. (1978). Raman spectra of possible corrosion products of iron. *Appl. Spectrosc.* 32(6), 532-535.
